# Recurrent Catatonia in the Setting of Sickle Cell Pain Crises: Case Report

**DOI:** 10.1155/crps/9882696

**Published:** 2026-08-24

**Authors:** Mary Nance, Miracline Ebijoyeldhas, Mariam Khan, Kathleen A. Crapanzano

**Affiliations:** ^1^ Department of Psychiatry, LSU Health New Orleans School of Medicine, Baton Rouge Regional Campus, Baton Rouge, Louisiana, USA

**Keywords:** case report, pain crisis, recurrent catatonia, sickle cell

## Abstract

Catatonia is a neuropsychiatric syndrome with motor and behavioral symptoms, including mutism, negativism, posturing, stereotypy, and hypoactivity. Although previously linked primarily to psychiatric illness, a variety of medical conditions and substance intoxication or withdrawal have also been implicated. It is important to identify all potential underlying drivers of catatonia and treat them along with the catatonia itself for the best outcome. We present the case of a 48‐year‐old female with a psychiatric history of bipolar disorder and past medical history of sickle cell disease who has frequently presented to the hospital with sickle cell pain crises. She has had at least 11 incidences of sickle cell pain crisis with a comorbid episode of catatonia, a condition that has not been previously well reported with the development of recurrent catatonia in the literature. Improvement of symptoms in this patient requires management of both her pain crisis and benzodiazepines for treatment of catatonia.

## 1. Introduction

Catatonia is a neuropsychiatric syndrome that occurs in up to 20% [1] of psychiatric inpatients and up to 8% of inpatients on medical units [1]. Catatonia is characterized by a “marked psychomotor disturbance that may involve decreased motor activity, decreased engagement during the interview or physical examination, or excessive and peculiar motor activity.” [2] Per DSM‐V diagnostic criteria, catatonia involves a clinical picture that is dominated by three or more of the following: stupor, catalepsy, waxy flexibility, mutism, negativism, posturing, mannerism, stereotypy, agitation, grimacing, echolalia, or echopraxia [2]. When left untreated, catatonia can become life‐threatening due to autonomic instability, and further potential complications include pressure wounds, deep vein thromboses, and malnutrition from not eating. Catatonia has been attributed to both underlying psychiatric and medical etiologies.

Over the last 150 years, our understanding of catatonia has changed drastically. DSM‐5 moved catatonia from a subtype of schizophrenia with psychomotor disturbances to its own diagnostic entity. Many recent studies have explored the neurobiological aspects of catatonia. The suspected neurotransmitter systems that are implicated include gamma‐aminobutyric acid (GABA) and glutamate. More recent literature aims to link catatonia to neuroinflammation [3] due to a correlation with decreased serum iron levels, which serves as an indicator of inflammation [4]. Furthermore, structural imaging has linked catatonia to “widespread reductions in gray‐matter volume, including the orbitofrontal, cingulate, and visual cortices and the insula when compared to healthy individuals.” [4]

First‐line treatment for catatonia is the use of benzodiazepines, often with a “lorazepam challenge.” [5] Lorazepam often provides symptomatic relief, but full resolution is dependent on treatment of the underlying cause—whether medical, psychiatric, or both. One study found that in the hospital setting, 20% of catatonia diagnoses were due to an underlying medical condition. In a switch from the traditional view of catatonia as a subtype of schizophrenia, in acute medical and surgical settings, medical catatonia made up more than half of the cases of consults for catatonia. This increased to 80% when the population was older adults and critically ill patients seen by consult psychiatry services with catatonic symptoms [6]. In a systematic review of causes of medical catatonia, two‐thirds of the reported cases involved CNS‐specific pathologies, including encephalitis, neural injury, developmental disorders, structural brain pathology, or seizures [6]. However, there are innumerable conditions that have been associated with catatonia, including hyponatremia [7], B12 deficiency [8], and many others.

The prevalence of recurrent catatonia is difficult to discern from the literature despite many case reports. Recent reports vary from 1/3 to almost 50% [9, 10]. Recurrent catatonia can arise from both medical and psychiatric causes and is commonly reported with recurrent mood and psychotic episodes [11]. We present a case of recurrent catatonia in the context of a person with underlying bipolar disorder and sickle cell disease whose catatonic episodes are frequently associated with pain crises, necessitating treatment of both underlying medical and psychiatric conditions.

## 2. Case Presentation

Patient X is a 49‐year‐old African American female with a past medical history of sickle cell disease who was initially admitted to our psychiatric unit in 2014. She was diagnosed with bipolar disorder and discharged on quetiapine 300 mg at night and lamotrigine 100 mg twice a day. Prior to her worsening sickle cell disease and development of a psychiatric disorder, she was able to work as a nurse for 5 years after completing nursing school. Her family history includes an unspecified mental illness in her father and depression in an unspecified family member. Her next encounter with our service was in December 2018. At that time, she presented in a sickle cell crisis with additional catatonic symptoms of rigidity, mutism, negativism, agitation, grimacing, and minimal interactions with others. She also had periods of agitation and repetitive language. Her hemoglobin on admission was 8.8 g/dL. During this admission, neurology was consulted, and an extensive workup including lumbar puncture, EEG, and MRI was completed. On EEG, there were “few isolated epileptiform spikes noted in bilateral posterior parietal leads over the course of the study but no sustained epileptiform activity. This may represent residual cortical irritability from the prior subdural hematoma.” A subdural hematoma was seen on MRI, which was sustained from a fall approximately 1 month prior and was also likely the cause for elevated protein in her CSF, which was otherwise normal. Her MRI also showed diffuse medullary expansion of the cranium, likely secondary to sickle cell disease. However, it should be noted that there was no evidence of a subdural hematoma on imaging at this time. Her lamotrigine was increased by neurology due to concern for seizures. She was treated with 1 mg of intravenous lorazepam for catatonic symptoms, which improved. She was then transferred to the inpatient psychiatric unit, where her symptoms continued to improve, and she was able to describe feeling depressed. Her discharge medications included lorazepam 0.5 mg three times a day, quetiapine 300 mg at bedtime, and mirtazapine 15 mg at bedtime. At that time, however, she was noted to be in sickle cell crisis with a hemoglobin of 6.6 g/dL and was transferred back to the medical unit, where she received two units of packed red blood cells prior to discharge home. Subsequent imaging demonstrated the resolution of the previously mentioned subdural hematoma.

Since her discharge in January 2019 until December 2025, she had a significant number of ER presentations at our facility, resulting in 24 medical admissions, two psychiatric admissions, and two admissions where she was admitted medically or psychiatrically and then transferred to the other within our facility. Of the 24 medical admissions, 20 were for pain crises or low hemoglobin, and the remaining four were for anaphylaxis, cellulitis, encephalopathy, and an intentional overdose. Only two of her psychiatric admissions were not followed or preceded by a medical admission. During at least 15 admissions, she was noted to be catatonic at some point during her stay. Out of those 15 admissions, she was also at some point treated for sickle cell crisis during 11 of them. Primary teams were inconsistent in consulting psychiatry for management of catatonia, resulting in incomplete details regarding her condition. It was also noted during chart review that there were times she was restarted on her home medications, including lorazepam, and improvement was noted without the involvement of the psychiatry service. A summary of her hospitalizations is available in Table 1.

**Table 1:** Summary of hospitalizations from January 2019 until December 2025.

Admission	Admit type	Admit	Discharge	Psych consult during medical admission?	Psych meds at presentation	Discharge medications	Presenting symptoms	Sickle cell crisis?	Catatonic?	Symptoms or initial BF score	Lorazepam challenge and dose	Duration to catatonia resolution?	Blood transfusion?	Key metabolic labs (Na, K, Ca, Mg, B12, thyroid function, renal/hepatic panels)
1	Medical, psychiatric, medical	27‐12‐2018	28‐01‐2019	Yes	Lamotrigine 150 mg BID, quetiapine 300 mg, mirtazapine 15 mg	Lorazepam 0.5 mg TID, mirtazapine 15 mg, quetiapine 300 mg, lamotrigine 150 mg BID	Sickle cell related pain	Yes	Yes	Mutism, impulsivity, negativism	No	14 days after transfer to psych	1u	CPK 201, WBC 13, T. Bili 2.2
2	Psychiatric	04‐02‐2019	14‐02‐2019	—	Lorazepam 0.5 mg TID, mirtazapine 15 mg, quetiapine 300 mg, lamotrigine 150 mg BID	Lorazepam 1 mg TID, quetiapine 200 mg, haloperidol 2.5 mg BID	Noncompliance with psychotropic medication, catatonia	No	Yes	Mutism, staring, rigidity, posturing	Yes, 1 mg IV and 2 mg IM lorazepam in ED with some improvement	4 days	None	Cr 0.55
3	Medical	24‐08‐2019	03‐09‐2019	No	Lorazepam 1 mg TID, quetiapine 250 mg	Quetiapine 250 mg, lorazepam 1 mg TID	Generalized pain consistent with her typical vaso‐occlusive crises	Yes	No	—	N/A	N/A	1u	INR 4.6
4	Medical	29‐11‐2019	07‐12‐2019	Yes	Quetiapine 250 mg, lorazepam 0.5 mg TID	Quetiapine 250 mg, lorazepam 0.5 mg TID	Diffuse body pains	Yes	Yes	Mutism, staring, stereotypy	Yes, 2 mg IV with improvement	1 day	None	WBC 18.8
5	Medical, psychiatric, medical, psychiatric	17‐12‐2019	13‐02‐2020	Yes	Quetiapine 250 mg, lorazepam 0.5 mg QID PRN	Haloperidol 2 mg/mL 0.5 mLs by mouth TID, lorazepam 0.5 mg BID, mirtazapine sol‐tab 15 mg	Altered mental status, agitated behavior	Yes	Yes	Mutism, grimacing, posturing	Yes, 2 mg IV lorazepam with improvement	5 days after transfer to psych	2u	T. Bili 5.1, Cr 0.54
6	Medical	15‐03‐2020	20‐03‐2020	No	Haloperidol 2 mg/mL 0.5 mL TID, quetiapine 50 mg	Quetiapine 50 mg	Generalized pain consistent with her typical vaso‐occlusive crises	Yes	No	—	No	N/A	None	WBC 15.3, Ca 8.2
7	Medical	21‐09‐2020	02‐10‐2020	No	Lorazepam 0.5 mg QID PRN, mirtazapine sol‐tab 15 mg, quetiapine 50 mg	Lorazepam 0.5 mg QID PRN, melatonin 10 mg, mirtazapine sol‐tab 15 mg, quetiapine 50 mg	Sickle cell pain crisis/DVT	Yes	No	—	No	N/A	None	WBC 18, LDH 563
8	Medical	06‐10‐2020	13‐11‐2020	Yes	Mirtazapine sol‐tab 15 mg, quetiapine 50 mg, lorazepam 0.5 mg QID PRN	Haloperidol 1 mg BID, lorazepam 1.5 mg TID, melatonin 10 mg, mirtazapine sol‐tab 15 mg	Altered mental status	Yes	Yes	10	Yes, 2 mg IM required continued titration for improvement	Day before dc	None	H/H 9.5/30.3
9	Psychiatric, medical	17‐11‐2020	28‐11‐2020	Yes	Haloperidol 1 mg BID, lorazepam 1.5 mg TID, mirtazapine sol‐tab 15 mg	Haloperidol 1 mg BID, lorazepam 1 mg QID, mirtazapine sol‐tab 15 mg, hydroxyzine 25 mg TID PRN	Abnormal labs and aggressive behavior	Yes	Yes	Mutism	No, resumed home ativan with improvement	4 days	None	Ca 8.4, T. Bili 2.5, Na 131
10	Medical	28‐07‐2022	03‐08‐2022	Yes	Haloperidol 2 mg, atarax 25 mg QID PRN, lorazepam 2 mg QID PRN, quetiapine 100 mg	Discharged to another inpatient psychiatric hospital	AMS, intentional overdose	No	Yes	Mutism, staring, stupor	No, resumed home ativan with improvement	Day of dc	1u	K 5.2. ALT 71
11	Medical	06‐09‐2022	11‐09‐2022	Yes	Bupropion SR 100 mg BID, venlafaxine XR 75 mg	Bupropion SR 100 mg BID, venlafaxine XR 75 mg, lorazepam 1 mg TID	Sickle cell pain crisis and catatonia	Yes	Yes	15	Yes, 1 mg IV with improvement	3 days	1u	WBC 15.7
12	Medical	21‐04‐2023	25‐04‐2023	No	Bupropion 100 mg, haloperidol 2 mg BID, atarax 25 mg PRN, lorazepam 0.5 mg BID, quetiapine 200 mg, venlafaxine 75 mg	Bupropion 100 mg BID, haloperidol 2 mg, hydroxyzine 25 mg TID PRN, lorazepam 1 mg TID, quetiapine 200 mg, venlafaxine 37.5 mg	Told to come in due to dec Hb on recent lab hemoglobin of 5.4	Yes	No	—	No	N/A	1u	WBC 11.3
13	Medical	02‐06‐2023	05‐06‐2023	No	Bupropion 100 mg BID, haloperidol 2 mg, hydroxyzine 25 mg TID PRN, lorazepam 1 mg TID, quetiapine 200 mg, venlafaxine 37.5 mg	Bupropion 100 mg BID, haloperidol 2 mg, hydroxyzine 25 mg TID PRN, lorazepam 1 mg TID, quetiapine 200 mg, venlafaxine 37.5 mg	Uncontrolled sickle cell pain	Yes	No	—	No	N/A	1u	T. Bili 4.8, Ca 8.5
14	Medical	13‐06‐2023	22‐06‐2023	No	Bupropion 100 mg BID, haloperidol 2 mg, hydroxyzine 25 mg QID PRN, lorazepam 1 mg TID, quetiapine 200 mg, venlafaxine 37.5 mg	Bupropion 100 mg BID, haloperidol 2 mg, hydroxyzine 25 mg QID PRN, lorazepam 2 mg TID, quetiapine 200 mg, venlafaxine 37.5 mg	Pain crises, fatigue	Yes	Yes	Lethargic, limited interaction, not seen by psychiatry	Restarted home medications and titrated to 2 mg TID	7 days, 2 days before discharge	1u	T. Bili 2.8
15	Medical	09‐12‐2023	15‐12‐2023	No	Bupropion 100 mg BID, haloperidol 2 mg, hydroxyzine 25 mg TID PRN, lorazepam 2 mg TID PRN, quetiapine 400 mg, venlafaxine 37.5 mg	Bupropion 100 mg BID, haloperidol 2 mg, hydroxyzine 25 mg TID PRN, lorazepam 2 mg TID PRN, quetiapine 400 mg, venlafaxine 37.5 mg	Shortness of breath	Yes	No	—	No	N/A	3u	Ca 8.4
16	Medical	11‐01‐2024	14‐01‐2024	No	Bupropion 100 mg BID, haloperidol 2 mg, hydroxyzine 25 mg TID PRN, lorazepam 2 mg TID PRN, quetiapine 400 mg, venlafaxine 37.5 mg	Bupropion 100 mg BID, haloperidol 2 mg, hydroxyzine 25 mg TID PRN, lorazepam 2 mg TID PRN, quetiapine 400 mg, venlafaxine 37.5 mg	Altered mental status and hypoxia	Encephalopathy	No	—	No	N/A	None	Ca 8.3, WBC 12.6
17	Medical	08‐02‐2024	09‐02‐2024	No	Bupropion 100 mg BID, haloperidol 2 mg, hydroxyzine 25 mg TID PRN, lorazepam 2 mg TID PRN, quetiapine 400 mg, venlafaxine 37.5 mg	Bupropion 100 mg BID, haloperidol 2 mg, hydroxyzine 25 mg TID PRN, lorazepam 2 mg TID PRN, quetiapine 400 mg, venlafaxine 37.5 mg	Allergic reaction	Anaphylaxis due to almonds	No	—	N/A	N/A	No because of anaphylaxis	WBC 20.4, T. Bili 4.5
18	Medical	28‐03‐2024	30‐03‐2024	No	Bupropion 100 mg BID, haloperidol 2 mg, hydroxyzine 25 mg TID PRN, lorazepam 2 mg TID PRN, quetiapine 400 mg, venlafaxine 37.5 mg	Bupropion 100 mg BID, haloperidol 2 mg, hydroxyzine 25 mg TID PRN, lorazepam 2 mg TID PRN, quetiapine 400 mg, venlafaxine 37.5 mg	Worsening mouth pain, body pain	Yes	No	—	N/A	N/A	1u	Ca 8.4, T. Bili 4
19	Psychiatric, medical	02‐04‐2024	22‐04‐2024	Yes	Lorazepam 2 mg TID PRN, hydroxyzine 25 mg TID PRN, haloperidol 2 mg, quetiapine 400 mg	Lorazepam 1 mg QID, quetiapine 250 mg, venlafaxine 37.5 mg	Screaming in pain, exhibiting a nosebleed, facial swelling, and difficulty breathing, concern for benzodiazepine withdrawal seizure	Yes	Yes	19	Restarted home regimen	Until 4/21	2u during admission	T. Bili 3.6, Cr 0.53, Ca 8.7
20	Medical	17‐05‐2024	19‐05‐2024	Yes	Lorazepam 2 mg TID PRN, quetiapine 250 mg, venlafaxine 37.5 mg	Hydroxyzine 25 mg TID PRN, lorazepam 2 mg TID PRN, quetiapine 250 mg, venlafaxine 37.5 mg	Allergic reaction, face swelling	Yes	Yes	7	Discharged with residual symptoms, was not restarted on home dose until last day of admit	Catatonic sx upon dc	1u	T. Bili 4.4, k 2.9
21	Medical	10‐11‐2024	18‐11‐2024	No	Lorazepam 2 mg qAM, 1 mg afternoon and 2 mg hs, hydroxyzine 25 mg TID PRN, quetiapine 400 mg, venlafaxine 37.5 mg	Hydroxyzine 25 mg TID PRN, lorazepam 2 mg qAM, 1 mg afternoon and 2 mg hs, quetiapine 400 mg, venlafaxine 37.5 mg	Bilateral lower leg pain	Yes	No	—	No	N/A	1u	Cr 0.44, Ca 8.5
22	Medical	19‐11‐2024	28‐11‐2024	Yes	Lorazepam 2 mg qAM, 1 mg afternoon and 2 mg hs, hydroxyzine 25 mg TID PRN, quetiapine 400 mg, venlafaxine 37.5 mg	Hydroxyzine 25 mg TID PRN, lorazepam 2 mg qAM, 1 mg afternoon and 2 mg hs, quetiapine 400 mg, venlafaxine 37.5 mg	Sickle cell pain crisis	Yes	Yes	Mutism, posturing, grimacing, staring	2 mg IM x1, followed by restarting home dose	2 days	1u	Cr 0.45, Ca 7.7
23	Medical	26‐01‐2025	01‐02‐2025	Yes	Quetiapine 400 mg, venlafaxine 75 mg, hydroxyzine 25 mg TID PRN, lorazepam 2 mg TID	Hydroxyzine 25 mg TID PRN, lorazepam 2 mg TID, quetiapine 400 mg	Sickle cell pain crisis	Yes	Hypomania	0	N/A	N/A	1.7u	Ca 7.9
24	Medical	10‐02‐2025	14‐02‐2025	No	Hydroxyzine 25 mg TID PRN, lorazepam 2 mg TID, quetiapine 400 mg	Hydroxyzine 25 mg TID PRN, lorazepam 2 mg TID, quetiapine 400 mg	Generalized pain about 7/10 severity	Yes	No	—	N/A	N/A	1u	Cr 0.5, Ca 8.4
25	Medical	06‐03‐2025	08‐03‐2025	No	Hydroxyzine 25 mg TID PRN, lorazepam 2 mg TID, quetiapine 400 mg	Hydroxyzine 25 mg TID PRN, lorazepam 2 mg TID, quetiapine 400 mg	Chest tightness for couple of days with associated shortness of breath of 2 days duration, nosebleed	Yes	No	—	N/A	N/A	2.5u	Cr 0.51, Ca 8.3
26	Medical	20‐03‐2025	23‐03‐2025	No	Hydroxyzine 25 mg TID PRN, lorazepam 2 mg TID, quetiapine 400 mg	Hydroxyzine 25 mg TID PRN, lorazepam 2 mg TID, quetiapine 400 mg	Neck pain with swelling and chest pain	Yes	Yes	Mutism	Continued home PO dose with improvement in symptoms	N/A	1u	Total bilirubin 2.6
27	Medical	18‐04‐2025	21‐04‐2025	No	Haloperidol 10 mg hs, quetiapine 400 mg hs, lorazepam 2 mg TID, venlafaxine 75 mg, hydroxyzine 25 mg TID PRN	Haloperidol 10 mg hs, hydroxyzine 25 mg TID PRN, lorazepam 2 mg TID, quetiapine 400 mg	Bilateral leg and knee pain worse on the right side	No	No	—	No	N/A	1u	Alk phos 284
28	Medical	27‐04‐2025	29‐04‐2025	No	Haloperidol 10 mg hs, lorazepam 2 mg TID, quetiapine 400 mg	Haloperidol 10 mg hs, lorazepam 2 mg TID, quetiapine 400 mg	Bilateral lower extremity and back pain typical of her sickle cell pain crisis	Yes	No	—	No	N/A	No	Ca 8.3, Cr 0.42, total bilirubin 1.6
29	Psychiatric	20‐05‐2025	03‐06‐2025	No	Lorazepam 2 mg TID, quetiapine 400 mg nightly and 50 mg BID prn for anxiety	Lorazepam 2 mg QID daily, quetiapine 100 mg nightly	Mood fluctuations described as agitation/"violent outbursts” to unresponsiveness/catatonia	No	Yes	Mutism, staring	Was started on 2 mg PO TID	12 days	No	Ca 7.8, bilirubin 2.5
30	Medical	18‐07‐2025	24‐07‐2025	No	Lorazepam 2 mg QID daily, quetiapine 400 mg nightly	Lorazepam 2 mg QID daily, quetiapine 400 mg nightly	Pain all over, shortness of breath	Yes	No	—	No	N/A	1u	Cr 0.54
31	Medical	25‐07‐2025	29‐07‐2025	No	Lorazepam 2 mg QID daily, quetiapine 400 mg nightly	Lorazepam 2 mg QID daily, quetiapine 400 mg nightly	Whole body aches, swelling in legs	Yes	No	—	No	N/A	1u	TSH 8.824
32	Medical	28‐11‐2025	02‐12‐2025	Yes	Lorazepam 2 mg QID daily, quetiapine 400 mg nightly	Lorazepam 2 mg QID daily, quetiapine 400 mg nightly	Sickle cell pain crisis, catatonic state	Yes	Yes	15	Continued home PO dose	Once pain medicine was received	0.75u	TSH 7.526

Of note, between November 2020 and July 2022, she did not present to the emergency department or require admission to the hospital. During that time, she was on hospice care for management of her symptoms, and we presume that they were able to maintain her fluid status and provide adequate pain management and benzodiazepines to manage her symptoms. She was discharged from hospice in March of 2022, as she no longer met hospice criteria. Her next presentation was in July 2022, for an intentional overdose due to not wanting to be a burden on her family anymore. Following this admission, she continued to have repeated presentations as described above until the present day.

## 3. Discussion

This unusual case of a woman with sickle cell disease with both recurrent pain crises and catatonic episodes that appear to be associated has not been previously well described. We could find no other published case reports demonstrating an association between recurrent catatonia symptoms and sickle cell disease, although there are a few reports of single episodes of catatonia occurring in patients with a sickle cell diagnosis, though these do not aim to link the two directly [12–14]. While there are certainly many confounding variables in our patient, we propose that the effects of her sickle cell and bipolar disorder have contributed in an additive way to the development of catatonia symptoms.

We have several theories as to how the two could be linked. First, hypoxia due to vaso‐occlusive crises could be driving the initiation of catatonic symptoms. Cerebral hypoxic events are known to result in catatonia symptoms [15]. Her most recent brain MRI in April 2024 showed scattered hyperintense lesions in the white matter on T2/FLAIR that likely represent chronic small vessel ischemic disease. We suspect that these chronic and repeated insults, in addition to potential acute hypoxia, have created an environment that could lead to catatonic symptoms. There are few case reports in the literature describing catatonia in cases of delayed posthypoxic leukoencephalopathy [15, 16] and following cerebrovascular accident [17], suggesting an underlying mechanism between hypoxia and catatonia.

The patient also carries a diagnosis of bipolar disorder, which has an association with recurrent catatonia [11]. However, she has had multiple presentations for symptoms of sickle cell crises and catatonia that did not require further inpatient psychiatric treatment for the management of bipolar symptoms. While her bipolar disorder and any acute episode of depression or mania could contribute to the development of catatonia in an additive sense, they do not explain her recurrent episodes in the absence of a mood episode. Her primary presenting symptoms seem to be those of catatonia in the context of a sickle cell pain crisis. Additionally, while we do not have evidence in each sickle cell crisis presentation of catatonia, it should be noted that each time psychiatry was consulted during an admission, the psychiatry consult service noted catatonic symptoms (except for one presentation during which she was hypomanic). Due to psychiatry inconsistently being involved in her care, the catatonia symptoms may have been overlooked, not documented, or missed by our medical colleagues during the other admissions. Of note, the patient did not present with any catatonic or sickle cell crisis episodes while she was on home hospice that required admission between November 2020 and July 2022. It is unknown if she was symptomatic during this period and managed at home or whether good supportive care alleviated her pain crisis and catatonia symptoms entirely.

This case demonstrates a complex interplay between medical and psychiatric disease, culminating in recurrent catatonic symptoms that have necessitated long‐term use of benzodiazepines. Attempts to wean benzodiazepines have occurred but resulted in the reemergence of catatonia symptoms, and she has continued to require escalating doses even in the outpatient setting. Electroconvulsive therapy (ECT) has been considered at various times throughout her illness course. However, our facility does not offer ECT, and prior inpatient transfers were unable to be completed. On an outpatient basis, she did see a provider who offered ECT but ultimately has not had ECT as of the time of this article.

It is nearly impossible to tease out the symptoms of catatonia from this patient’s underlying medical and psychiatric disorders. She has also been on daily lorazepam for several years, further introducing a component of potential GABA withdrawal if there are missed doses. However, after a very detailed review of the chart and the literature, it is likely that both her medical and psychiatric conditions have led to a chronic presentation consistent with recurrent catatonia. She does best when on chronic maintenance benzodiazepines and quetiapine and with adequate treatment of her sickle cell (often in the form of opioid medications, hydration, and adequate oxygenation, as she at times needs supplemental oxygen during hospital stays). In order to treat catatonia, treating the underlying cause is also essential, and as such, she has been maintained on antipsychotics as well for the treatment of her underlying bipolar disorder. She did not present with autonomic instability or hyperthermia that would raise suspicion for neuroleptic malignant syndrome, and her often swift improvement with benzodiazepines lends support to a diagnosis of catatonia.

Overall, we propose that the chronic neurologic insults from sickle cell crises and hypoxia have led to higher susceptibility to catatonia, in addition to having bipolar disorder, which itself carries a risk of catatonia. This case highlights the importance of managing both psychiatric and medical comorbidities and that one cannot be neglected in favor of treating the other. This case serves as a reminder to consider the whole patient when treating for symptoms that may often present as primarily due to an underlying psychiatric etiology.

## Funding

No funding was received for this manuscript.

## Disclosure

No funding was received by the authors; rather, the work was performed as part of their employment by the LSU School of Medicine.

## Ethics Statement

This single‐patient case report was IRB‐exempt under institutional policy under the Louisiana State University IRB. The report was prepared in accordance with institutional ethical standards and the CARE guidelines.

## Consent

Written informed consent was obtained from the patient’s next of kin for the publication of this case report. All identifying patient information has been removed to protect patient privacy.

## Conflicts of Interest

The authors declare no conflicts of interest.

## Data Availability

Data sharing is not applicable to this article, as no datasets were generated or analyzed during the current study.
